# 1309. Incidence and Epidemiology of Invasive Pneumococcal Disease due to Serotype 3 in South-Central Ontario

**DOI:** 10.1093/ofid/ofab466.1501

**Published:** 2021-12-04

**Authors:** Allison McGeer, Agron Plevneshi, Kazi Hassan, Wayne Gold, Larissa Matukas, Tony Mazzulli, David Richardson, Reena Lovinsky, Irene Martin, Kevin Katz, Mahin Baqi, Sharon Walmsley, Christie Vermeiren, Altynay Shigayeva, Zoe Zhong

**Affiliations:** 1 Sinai Health System, Toronto, Ontario, Canada; 2 University of Toronto, Toronto, ON, Canada; 3 Unity Health, Toronto, Ontario, Canada; 4 William Osler Health System, Brampton, ON, Canada; 5 Scarborough Health Network, Scarborough, Ontario, Canada; 6 National Microbiology Laboratory, Public Health Agency of Canada, Winnipeg, MB, Canada; 7 North York General Hospital, Toronto, Ontario, Canada; 8 University Health Network, Toronto, Ontario, Canada; 9 Shared Health Laboratories, Toronto, Ontario, Canada; 10 Sinai health, Toronto, Ontario, Canada

## Abstract

**Background:**

In our population, the most common serotype (ST) of *S. pneumoniae* causing invasive pneumococcal disease (IPD) is now ST 3. We undertook an analysis of population based surveillance for IPD to examine the incidence and epidemiology of ST 3 disease over the last 25 years.

**Methods:**

The Toronto Invasive Bacterial Diseases Network has performed population-based surveillance for IPD in Toronto/Peel region (pop’n 4.5M) since 1995. All sterile site isolates of *S. pneumoniae* are reported to a central study laboratory, isolates are serotyped, and clinical and vaccination data are collected via patient and physician interview and chart review. Population data are obtained from Statistics Canada.

**Results:**

From 1995-2020, 11032 episodes of IPD occurred; 10015 had STs available, and 10484 clinical data. Overall, ST 3 comprised 9.2% of cases (N=931). Compared to other patients with IPD, those with ST 3 IPD were older (median age 65 vs. 58.5, P< .001), more likely to have underlying lung (22.7% v 16.0%, P< .0001) and cardiac (21.7 v 18.4, P=.02) disease and less likely to be immunocompromised (IC) (23.1% v 29.0% P< .0001). ST3 episodes were more likely to be pneumonia (81% v 65%), less likely to be bacteremia without focus (7.6% v 18.9%), and more likely to require ICU admission (42.3% v 25.1%) and to die (27.1% v 16.6%). In multivariable analysis, patients with ST 3 disease remained more likely to die (OR 1.65; 95%CI1.3-2.0). Over time, the proportion of patients with ST 3 IPD who were nursing home (NH) residents (18/171 in 1995-2000 vs. 4/215 in 2016-2020, P=.0002), and who were IC (46/169 in 1995-2000 vs 39/204 in 2016-2020, P=.007) decreased significantly; in IPD due to other STs, the proportion who were NH residents declined, but the proportion IC increased significantly. The case fatality rate (CFR) declined significantly in IPD due to ST3 but not other STs (Figure 1). Changes in incidence are shown in Figure 2.

Figure 2: Incidence of serotype 3 IPD over time, Toronto/Peel, 1995-2020

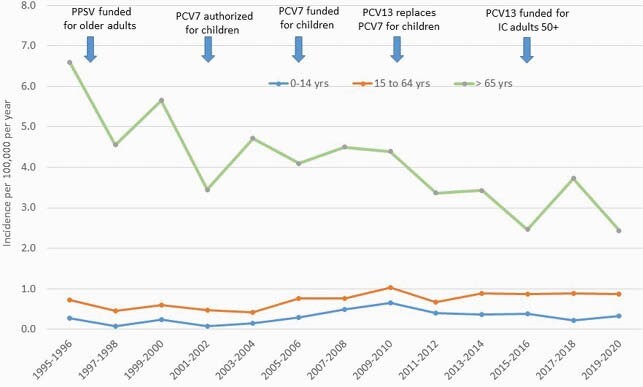

The incidence of ST3 IPD in children and adults under 65 did not change significantly from 1995/96 to 2019/20. In older adults, the annual incidence of disease declined from 4.98 per 100,000 per year in 1995-2000 to 3.53 per 100,000 per year in 2001-2010 (IRR 0.71, 95%CI 0.56-0.90), then to 2.23 per 100,000 per year in 2011-2020 (IRR compared to 2001-2010 0.63, 95%CI 0.50-0.79)

FIgure 2: Case fatality rate of IPD due to serotype 3 and other serotypes over time, 1995-2020, Toronto-Peel

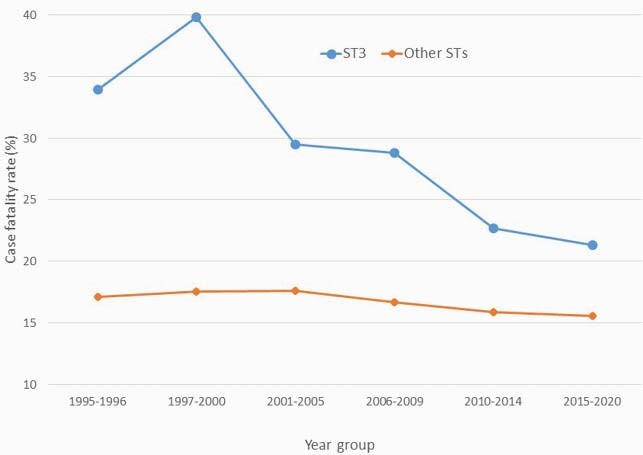

The case fatality rate of IPD due to ST3 declined from 37.6% (56/149) in 1995-2000 to 50/235 (21.3%) in 2015-2020 (P<.0001). The CFR in other serotypes did not change.

**Conclusion:**

The epidemiology of IPD due to ST3 has changed significantly over time and the CFR has declined. The incidence of ST3 disease in children and younger adults has not changed significantly, although the power to detect change is low in children. In older adults the incidence of ST3 disease declined significantly after PPV23 introduction in 1995/6 and again after PCV13 introduction for children.

**Disclosures:**

**All Authors**: No reported disclosures

